# Supplementary material for: Prioritization of Microorganisms Isolated from the Indian Ocean Sponge Scopalina hapalia Based on Metabolomic Diversity and Biological Activity for the Discovery of Natural Products
Source: Microorganisms. 2023 Mar 8;11(3):697. doi: 10.3390/microorganisms11030697 (PMC10057949; doi:10.3390/microorganisms11030697)
Supplement: Supplementary file 1 [file microorganisms-11-00697-s001.zip › microorganisms-2251309-supplementary.pdf]

Table S1. Batch mode use for data processing with MZMine 3.

1) Mass detection

Scan MS1 centroid ; noise level : $5^{E}2$
Scan MS2 centroid ; noise level : $0^{E}0$

2) ADAP Chromatogram Builder

Min groupe size in # of scans : 3
Group intensity threshold : $5^{E}2$
Min highest intensity : $5^{E}2$
Scan to scan accuracy : 0.0050 *m/z* or 15 ppm

3) Local minimum feature resolver

MS/MS scan pairing, retention time (RT) tolerance : 0.2 min ;
MS1 to MS2 precursor tolerance : 0.0080 *m/z* or 15 ppm
Chromatographic threshold : 90%
Minimum search range RT : 0.080 min
Minimum relative height : 30%
Minimum absolute height : $1^{E}0$
Min ratio of peak top/edge : 1
Min # of data points : 3

4) Duplicate peak filter

m/z tolerance : 0.005 *m/z* or 15 ppm
RT tolerance : 0.05 min

5) 13C isotope filter

m/z tolerance : 0.005 *m/z* or 15 ppm
RT tolerance : 0.05 min
Maximum charge : 3
Representative isotope most intense

6) Join aligner

m/z tolerance : 0.005 *m/z* or 15 ppm
Weight for *m/z* : 3
RT tolerance : 0.08 min
Weight for RT : 1

7) Feature list blank subtraction

Minimum # of detection in blanks : 1

8) metaCorrelate

RT : 0.07 min
Min height : $5^{E}2$
Intensity correlation threshold : $5^{E}2$
Correlation grouping
Feature height correlation

9) Ion identity molecular networking

m/z tolerance 0.008 *m/z* or 15 ppm
Min height $5^{E}2$

10) Peak filter

Area $5^{E}2$ – $1^{E}7$

11) Feature list rows filter

Feature with MS2 scan
Reset the feature number ID

12) Export feature list GNPS

Feature intensity : peak area
CSV export simple

Table S2. Summary table of peaks observed in the chromatographic profiles HPLC-CAD for each microbial extract.

Strain	Retention time	Height pA	UV max
Bacillus berkeleyi SH-137	24.45	11.05	NA
	27.365	19.48	NA
	31.834	34.15	275
	32.267	44.20	190
	33.892	48.80	190
	36.301	19.32	210
Bacillus paralicheniformis SH-02	15.319	34.21	222
	33.508	30.00	190
	34.344	13.31	NA
	35.506	70.90	190
	36.082	43.96	190
	37.738	166.04	190
Bacillus licheniformis SH-68	24.788	10.16	190
	34.357	27.05	NA
	35.541	97.00	190
	37.588	43.56	190
Chaetomium globosum SH-123	20.003	40.17	221
	21.748	16.01	221 / 266
	23.027	21.59	221
	24.457	52.8	221
	31.476	23.01	NA
	31.599	24.92	NA
	34.063	15.11	190
	37.14	35.88	190
	45.03	29.79	190
	45.878	121.56	282
Micromonospora chokoriensis SH-36	NA	NA	NA
Micromonospora citrea SH-89	37.248	17.59	190
Micromonospora echinospora SH-57	35.304	16.7	NA
Micromonospora fluostatini SH-82	9.95	16.52	NA
	10.812	47.84	190
	11.027	22.7	NA
	11.451	37.81	190
	11.763	73,38	NA
	12.333	44.23	190
	13.045	14.9	190
	14.065	25.29	190
	15.409	22.96	281
	18.963	148.8	NA
	19.34	18.93	NA
	20.72	63.22	248
	21.526	27.52	NA
	21.733	25.32	NA
	22.777	118.1	NA
	34.702	50.31	NA
	35.331	66.08	NA
Salinispora arenicola SH-78	13.422	15.56	292
	20.262	11.39	293
	20.769	14.37	290
	24.775	10.31	NA
	26.758	16.79	NA
	29.51	11.79	190
	34.5	14.73	NA

Figure S1. HPLC-CAD chromatogram of the standardized extract at 10 mg/mL of *Micromonospora fluostatini* SH-82's extract (red; 82 1S 14A) and the culture blank with medium A1 (blue; B 1S 14A)
(5 to 100% acetonitrile (ACN) for 30 min at a flow rate of 0.7 mL/min).

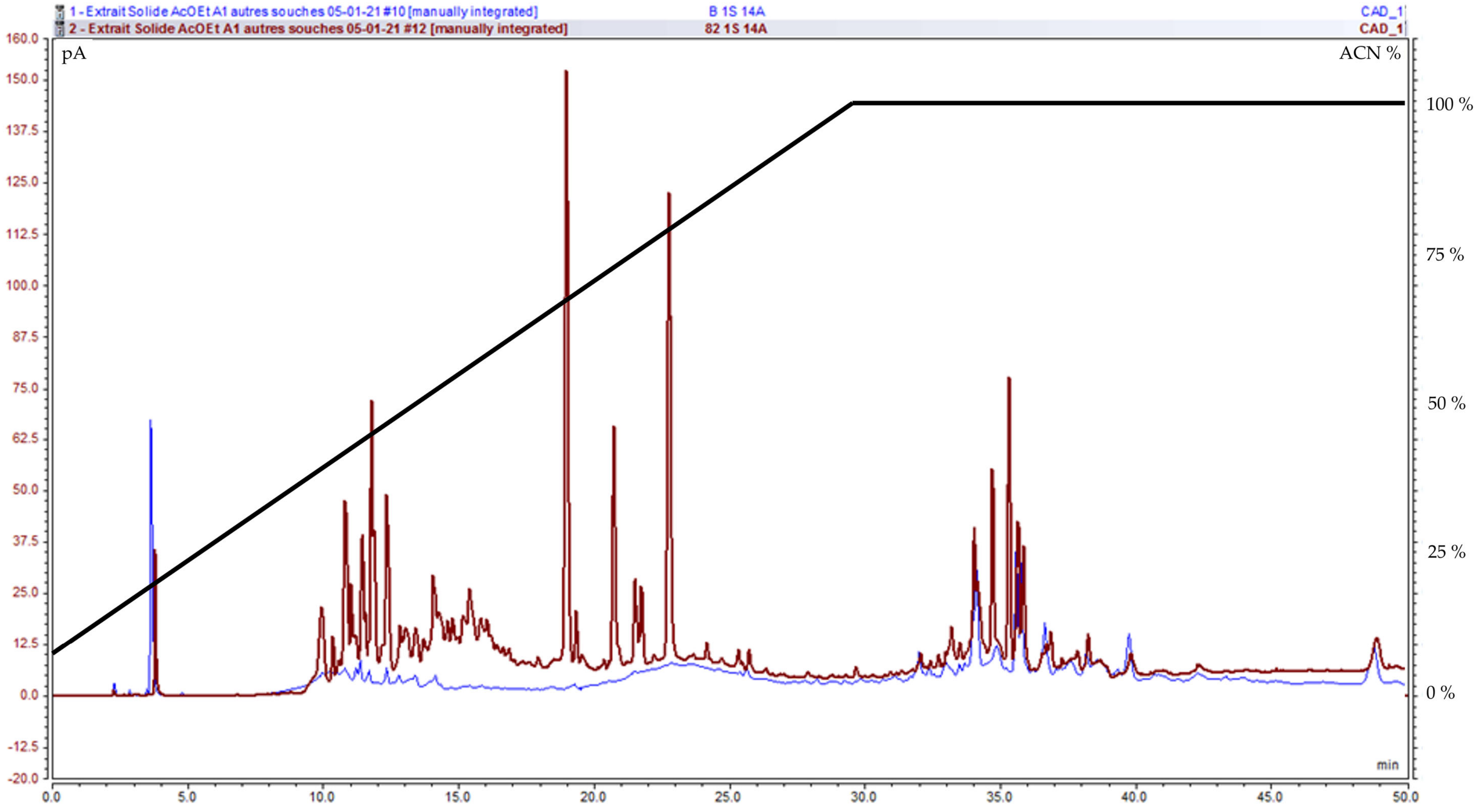

Figure S2. Molecular network representing chemical superclass of compounds in the microbial's extract. Nodes color describe the superclass, nodes shape indicated if the nodes were annotated or not, and nodes size is proportional to precursor ion intensity.

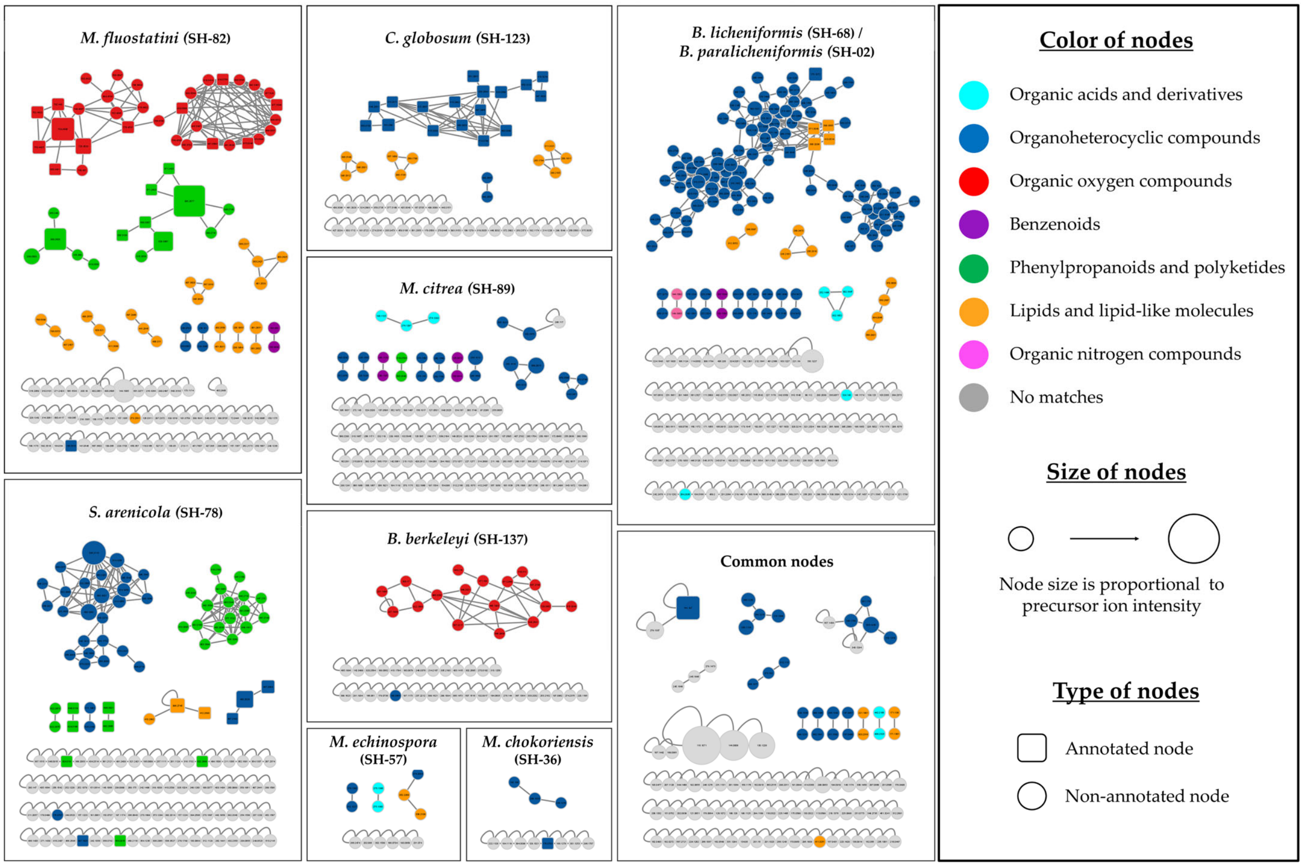

Figure S3. Cluster of *Micromonospora fluostatini* SH-82's extract (red node) containing dicharged and monocharged ions.

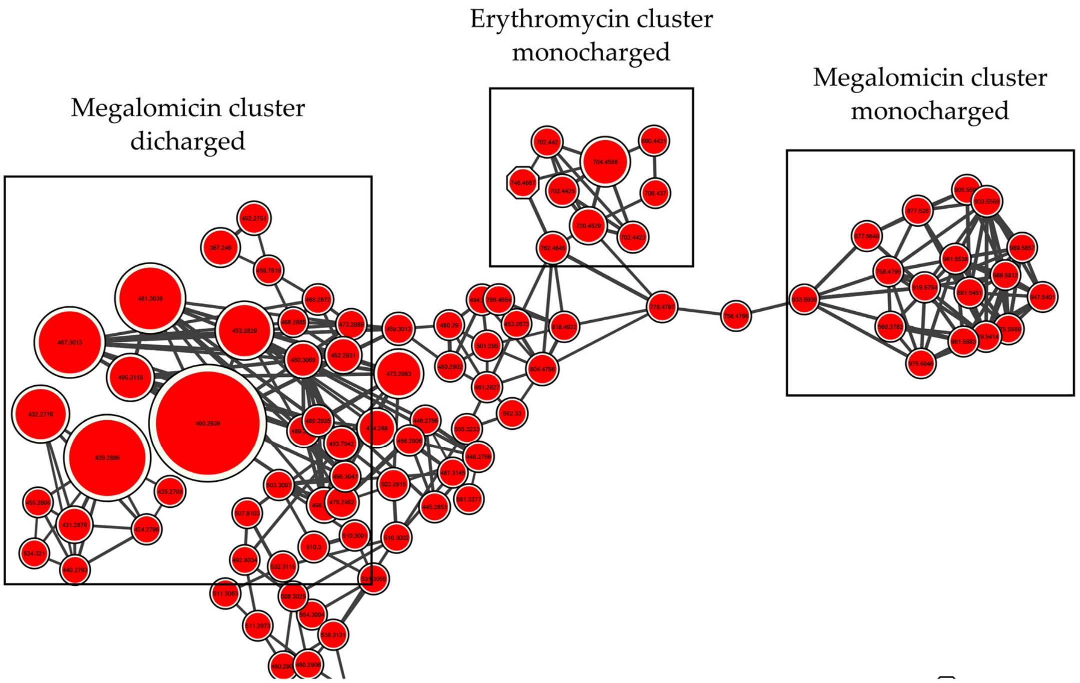

Figure S4. MS1 compounds C.1.20; 6-Deoxyerythronolide B ($C_{21}H_{38}O_6$)

m/z 369.2624; RT: 8.25 min; Best adduct: $[M-H_2O+H]^+$.

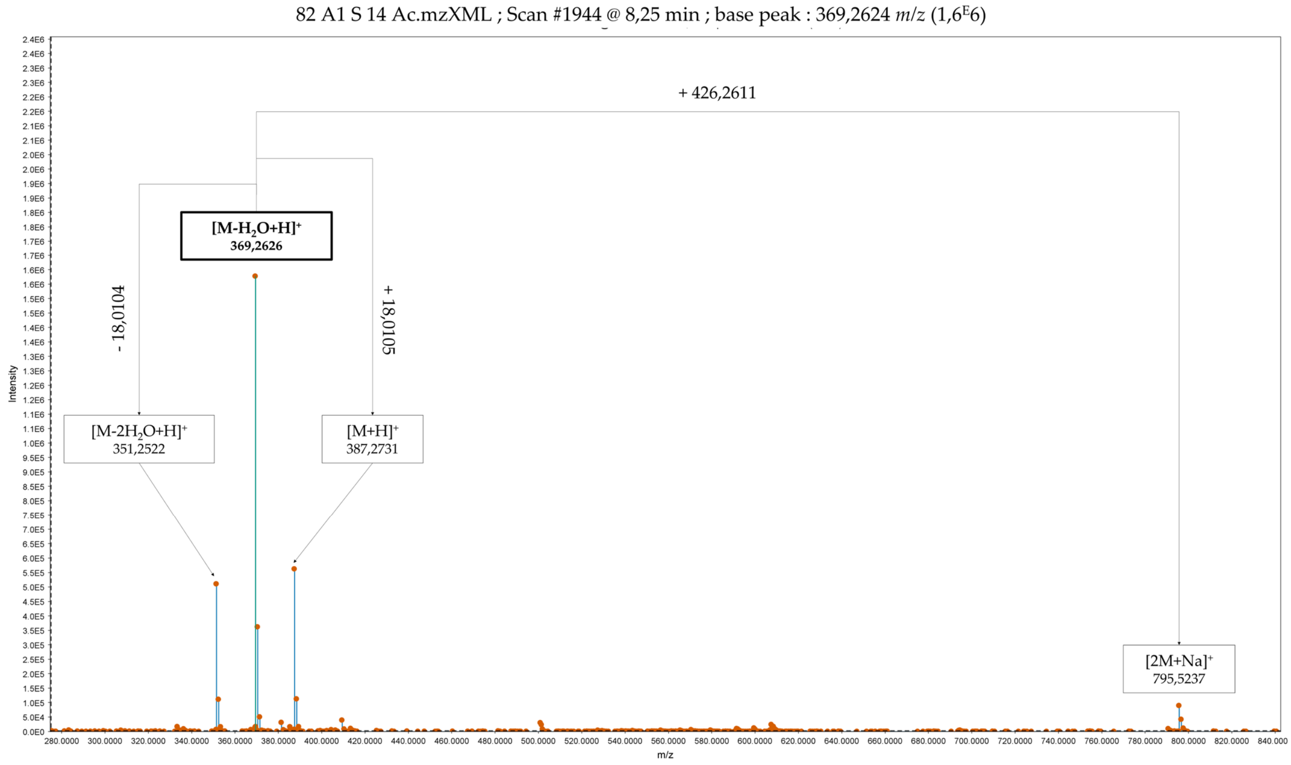

Figure S5. Cluster of *Salinispora arenicola* SH-78 (purple node) not accurately annotated.

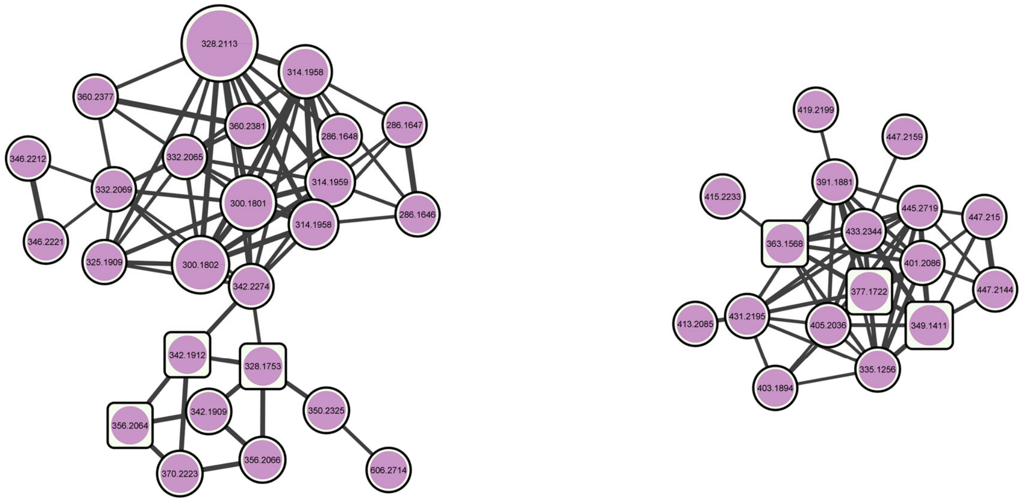

Table S3. Detailed table of chemical, biological and novelty scores for each strain studied.

Microbial strain	Chemical score (CS)					Biological score (BS)				Novelty score (NS)				
	HPLC-CAD score		IIMN score		Chemical score [1]	Cytotoxic activity score (BS_{CA})		Antiplasmodial activity score (BS_{AA})	Biological score [2]	LOTUS[3]	DNP[3]	NPatlas[3]	Average	Novelty score
	Score VP (CS_{VP})	Score MP (CS_{MP})	Score TN (CS_{TN})	Score UN (CS_{TN})		HCT-116 ($BS_{CA,HCT\text{-}116}$)	MDA-MB-231 ($BS_{CA,MDA\text{-}MB\text{-}231}$)							
Bacillus berkeleyi SH-137	3	2	3	2	**5**	4	1	4	**6.5**	0	0	0	0	**3**
Bacillus paralicheniformis SH-02	3	2	5	1	**6**	2	1	1	**2.5**	0	0	0	0	**3**
Bacillus licheniformis SH-68	2	1	5	1	**5**	1	1	1	**1.5**	42	45	18	35	**1**
Chaetomium globosum SH-123	4	2	3	3	**6**	4	4	5	**9**	294	196	143	211	**0**
Micromonospora chokoriensis SH-36	1	1	1	1	**2**	1	4	1	**3.5**	0	0	0	0	**3**
Micromonospora citrea SH-89	1	1	4	4	**5**	1	1	1	**1.5**	6	5	5	5	**3**
Micromonospora echinospora SH-57	1	1	1	1	**2**	3	2	1	**3.5**	64	41	33	46	**1**
Micromonospora fluostatini SH-82	5	5	5	5	**10**	2	1	5	**6.5**	0	0	0	0	**3**
Salinispora arenicola SH-78	3	1	5	5	**7**	5	5	5	**10**	59	31	41	44	**1**

[1] Chemical score, $CS = (CS_{VP} + CS_{MP} + CS_{TN} + CS_{UN}) / 2$
[2] Biological score, $BS = (BS_{CA,HCT\text{-}116} + BS_{CA,MDA\text{-}MB\text{-}231}) / 2 + BS_{AA}$
[3] Number of references on LOTUS, DNP, NPatlas
